# Our Vulnerable Dark Side—Two Laboratory Approaches

**DOI:** 10.3390/ijerph18083941

**Published:** 2021-04-09

**Authors:** Lena Lämmle, Matthias Ziegler

**Affiliations:** 1 Department of Psychology, Medical School Hamburg, Faculty of Human Sciences, MSH Medical School Hamburg, 20457 Hamburg, Germany; 2 Department of Psychology, Faculty of Life Sciences, Humboldt-Universität zu Berlin, 10117 Berlin, Germany; zieglema@hu-berlin.de

**Keywords:** Dark Triad, deliberate self-harm, vulnerability, white noise, electric shocks

## Abstract

The Dark Triad of personality has been associated with aggression against others as a reaction to perceived provocations. However, previous work has also shown that such responsive aggression even occurs if it means harming oneself. The first of two laboratory studies aimed to investigate whether this relation between the Dark Triad and self-harming behavior also occurs in situations where no others are affected but self-harm is likely. The second laboratory study considered two different settings in a within-participants design in order to analyze the stability of self-harming behavior and to what extent the Dark Triad constructs influence this behavior. The sample for study 1 consisted of 151 students (45.7% female) with a mean age of 21.40 years (*SD* = 2.19); the sample for study 2 consisted of 251 students (76.0% female) with a mean age of 22.21 years (*SD* = 3.90). Aside from the Dark Triad’s common core, depending on how self-harm was triggered (ego-threat (mainly narcissism), being alone with one’s own thoughts (mainly psychopathy), or reward condition (mainly Machiavellianism)), the Dark Triad traits differed in their responsiveness but were stable over the last two conditions, thereby suggesting a vulnerable side of the Dark Triad.

## 1. Introduction

The Dark Triad describes a cluster of dark personality traits situated within the larger Big Five network [1]. The three dark traits are narcissism, psychopathy, and Machiavellianism, which all share a malicious social character with a tendency to engage in antisocial behavior [2,3]. Past research has linked the Dark Triad traits to physical harm in real life, such as violent delinquency [4], or in lab situations, e.g., subjecting others to (white) noise in response to (perceived) provocation [5,6,7]. Moreover, there is initial evidence that the Dark Triad is not limited to other-directed but extends to self-directed aggressive and harmful behaviors [8]. A questionnaire-based and a laboratory study each revealed that individuals’ dark side is also associated with perceived victimization in workplace settings (e.g., in reaching one’s goals, job performance [9]) and willingness to subject oneself to white noise when others were also subjected to it. The use of white noise in laboratory studies was typically triggered by an upward comparison (i.e., comparison with a person who appears to be superior in certain ways) in combination with a monetary reward. Moreover, it has been shown that this behavior is related to the common core of the Dark Triad—the common core is assumed to be a tendency to maximize one’s own benefit, while disregarding, accepting, or malevolently provoking costs for others [10]—but also encompasses a smaller yet specific relation with the narcissistic part of the Dark Triad [8]. Thus, in line with previous findings that narcissism is associated with aggression in (non-)competitive settings [11], upward comparisons reflecting self-esteem threats [12] have been hypothesized to link narcissism [13] with self-aggression inflicted while engaging in aggression against perceived threats. However, it remains unknown whether upward comparisons provoke self-aggression in people scoring high on the dark core and narcissism if it is not combined with other-directed aggression (study 1).

Like narcissism [11], psychopathy has been identified as a risk factor for unprovoked aggression in the Tailor Aggression Paradigm: people high in psychopathy administered electric shocks without a previous physical provocation by their confederate [14]. Thus, the setting not only plays an important role in triggering aggressive behavior, but also in explaining it with respect to individual differences [15]. With regard to Machiavellianism, it is assumed that aggression towards others is determined by motives to establish social hierarchies or to assert power [16]. However, people high in Machiavellianism are usually reserved enough to realize that direct aggression rarely pays off [15] and only exploit others when it is profitable [17]. Thus, settings that do not satisfy any of those motivations might lead people high in Machiavellianism to seek to escape them, even at the cost of self-harming behavior. In line with this assumption is the finding by Wilson and colleagues [18] that being alone with one’s own thoughts for 15 min was experienced as so aversive that participants deliberately self-administered electric shocks [18]. Unfortunately, that study did not provide information on the role of personality traits in this behavior. Here, we modified Wilson and colleagues’ [18] design such that self-administered electric shocks were the way out of being alone with one’s own thoughts (study 2, condition 1). Based on prior research, we expected this escape option to be especially interesting for people high in psychopathy due to their risk of self-destructive behavior and their inability to delay gratification [17]. People scoring high on psychopathy but also on Machiavellianism can be motivated by material (e.g., money) and instrumental gain (e.g., power [19,20]). Thus, an experimental setting focusing on monetary rewards (study 2, condition 2) might also shed light on the self-harming side of Machiavellianism and psychopathy. Moreover, as the second study considered two different settings in a within-participants design, it allowed for analysis, firstly, of how stable self-harming behavior is and, secondly, of how much the Dark Triad’s common core and facets contribute to this behavior.

### 1.1. Paradigms for (Self-) Aggression

The association between the dark personality traits and other aggression has a long research tradition [21,22,23]. Recent lab-based aggression paradigms include, for example, the commonly used Competitive Reaction Time Task (sound-blast another person or receive a sound blast), a modified version [24] of the Tailor Aggression Paradigm (administer or receive electric shocks [11]), the Cold Pressor Task (choosing the time another individual has to hold their hand in ice water), the Hot Sauce Paradigm (choosing the amount of hot sauce another person has to consume), or the Uncomfortable Pose Task (choosing the time another individual has to hold an uncomfortable body position [24]). One reason why the Competitive Reaction Time Task is one of the most commonly used paradigms is Parrott and Giancola’s [25] assumption that the behavior is active (engaging in a behavior that results in harm to others) and direct (the perpetrator is easily identifiable to the victim, even when s/he does not exist because of a contrived interaction). In contrast to aggression directed at others, paradigms measuring laboratory-induced self-harming behavior are rather sparse. So far, to our knowledge, only an active and direct white noise paradigm has been used, in which inflicting white noise on others also meant having to endure white noise oneself [8]. Similarly, but without an opponent, the challenge of a disengaged mind that can be resolved by administering electric shocks [18] has also been studied in the context of self-harming behavior. Deliberate self-harming behavior is defined as a deliberate self-made injury without suicidal intent. A distinction is made between directly (e.g., burning, cutting, scratching) and indirectly harmful behavior (e.g., risky or indirect harmful behavior, such as reckless driving or unprotected sex with multiple partners [26]).

Thus, in study 1, where the research goal was to test whether self-harming behavior occurred even when no opponent was present, a paradigm combining multitasking with white noise was developed. Multitasking has been shown to have stress-inducing effects that are largely not perceived by humans [27]. In order to increase perceptibility, white noise serves to make the stress unpleasantly audible. As we additionally pursued the goal of shedding light on the question of whether self-harming behavior in different settings has differing behavioral affordances for the Dark Triad traits and their common core, two other settings for applying electric shocks were used in study 2. This follows the call by Hyatt and colleagues [11] to broaden the scope of contextual factors beyond competitive tasks for narcissism, extended to the Dark Triad. The change from white noise to electric shocks was conducted in order to build on the findings by Wilson and colleagues [18] on being alone with one’s own thoughts. At the same time, study 2 also follows Paulhus and colleagues’ [15] suggestion to acknowledge different settings in future research because each Dark Triad component exhibits an aggressive response to unique provocations.

### 1.2. Paradigms for Each Triad Member

Each Dark Triad trait displays subtle differences in the manifestation of aggression [28]; thus, it seems reasonable that this might also be true for self-harming behavior. While narcissism and Machiavellianism have been shown to be associated with hostility, psychopathy has been shown to be associated with actual physical aggression [22].

Ego threat (study 1) is sufficient [7] but not necessary to trigger aggression in people high in narcissism, as even non-competitive tasks seem to address constructs such as narcissism that are associated with antagonism [11]. This is because ego threat activates vulnerable narcissists’ sense of entitlement to maintain their grandiose self-views and seek out attention and praise [29]. Based on this and the previous finding that upward comparisons can trigger combined other- and self-harming behavior [8], the first study included the challenge of losing to an opponent. The underlying assumption is that people high in narcissism would engage in self-harm if this allowed the avoidance of self-esteem threats [12] and would try to reinforce their ego [30].

Nondescript rooms (i.e., being alone in a low stimulus room in which one is left with one’s own thoughts; condition 1 in study 2) combined with the possibility to self-administer electric shocks might be just what people high in psychopathy are waiting for: they can live out their impulsive side [31] and care little about their own physical safety [32]. Thus, given that psychopathy is a risk factor for unprovoked laboratory aggression towards others [14], it might also be a risk factor for unprovoked laboratory aggression towards the self. Additionally, as a sufficient number of electric shocks leads to release from the nondescript room, which is further accompanied by a monetary gain, this opportunity might also fit well with psychopaths’ inability to delay gratification [17].

People high in Machiavellianism adapt to different situations like a chameleon [33]. Their sensitivity to social contexts helps them switch between different tactics, make cool-headed decisions, and achieve what they strive for [33,34]. Thus, losing the option for strategic thinking [30] might threaten people high in Machiavellianism such that they are willing to harm themselves in order to escape the manipulation desert of a nondescript room.

Winning money for the highest number of electric shocks (condition 2 of study 2) potentially incorporates a competitive/ego-threat condition (narcissism [7]), unprovoked self-aggression and material gain (psychopathy [20]), as well as reward sensitivity (Machiavellianism [35]) and therefore, might be relevant for all three traits.

In sum, study 1 shed light on the question of whether upward comparisons provoke self-harming behavior in people scoring high on the dark core and narcissism if this behavior is not the by-product of other aggression. Study 2 provided information on the stability of self-harming behavior and to what extent the Dark Triad’s common core and its facets play a role therein. Thus, both studies gathered information on how three unique settings evoked forms of aggressive behavior specific to each triad member.

## 2. Materials and Methods for Study 1

### 2.1. Sample, Informed Consent and Ethical Approval

A total of 151 sport science students (45.7% female) with a mean age of 21.40 years (*SD* = 2.19) participated in the laboratory study. For capacity reasons, the sample size deviated from the required sample size of 241 participants (G*Power [36]) for an effect size of f^2^ = 0.046, which was found in a previous study on predicting deliberate self-harm with the Dark Triad [8]. Informed consent was obtained from all participants. They were informed that their participation was anonymous, completely voluntary, and that they could terminate their participation in the study at any time without any negative consequences. Participants were also informed that the collected information would only be used for the present study. Subjects were thanked and debriefed after participation. While planning the study, great care was taken to make sure that the study protocol adhered to the American Psychological Association’s Ethical Principles of Psychologists and Code of Conduct. Specific attention was paid to the participants’ well-being in light of the treatments applied.

### 2.2. Measures

Narcissism was assessed with the German version of the Narcissistic Personality Inventory (*NPI)* [37]. Its 40 forced-choice items (e.g., “I think I am a special person”) have been found to exhibit good psychometric properties [38]. The reliability estimate in the current data was acceptable (α = 0.82).

Psychopathy was assessed using a German translation of the Self-Report Psychopathy Scale (*SRP-III)* [39]. It consists of 64 items (e.g., “I am an impulsive person”) rated on a five-point scale, ranging from “strongly disagree” to “strongly agree”. Items were translated into German, translated back into English, and retranslated into German. Internal consistency was satisfactory (α = 0.88).

Machiavellianism was measured with the Machiavellianism Scale (*MACH-IV)* [40], a questionnaire consisting of 20 items (e.g., “The best way to handle people is to tell them what they want to hear”), using the same rating scale as the SRP-III. The translation procedure was the same. The instrument’s psychometric properties have been found to be good [41]. The overall internal consistency was acceptable (α = 0.70).

An automated version of the operation span task (AOSPAN) was used to assess working memory. This task has been shown to be a reliable and valid indicator in a wide array of research domains [42] and is considered a multitasking task [43].

Results for all variables used in the study can be found in Table 1.

### 2.3. Study Design

Before participating in the laboratory study, participants filled out an online survey in which the Dark Triad traits were assessed. Upon arriving at the laboratory, participants were reminded that the study is a stress-inducing working memory study. Participants were given the opportunity to choose between two processing forms. They could either choose a multitasking variant, which involved solving math problems correctly and quickly, while simultaneously trying to remember a series of letters. Otherwise, they could choose an alternative variant, in which the math problems to be worked on and the series of letters to be remembered appeared successively and could therefore be solved separately. Participants were told that, due to parallel processing, the multitasking variant could be expected to save time and that this was relevant because both processing time and number of errors would be assessed in the end.

However, participants were further informed that before they chose between the two variants, they needed to be aware that previous studies had found a stress-inducing effect of multitasking [27], such that it could be counted as self-harming behavior. They were informed that they should be further aware that the stress induced is typically not perceived by humans. For this reason, participants were asked to wear a heart rate monitor by the company Polar connected to a computer. This connection was claimed to be necessary in order to make the multitasking-induced stress clearly physically perceptible by means of white noise played through headphones. Thus, as one’s stress level increased during the multitasking variant, the white noise increased, up to a maximum of 75 dB (the maximum dB for music players in Germany is limited to 85 dB). Again, participants were informed that white noise counts as self-harming behavior. The maximum volume of 75 dB was then played to the participants as a listening sample, as long as they agreed. Participants were then asked to choose between the multitasking condition or the alternative variant.

Independently of their decision, participants then worked on the AOSPAN exercises in both variants and were then asked once again which variant they would like to choose. Afterwards, participants were instructed that their heart rate and test results would be compared to those of a randomly assigned participant who had already completed the study with two implications. Participants were told that, firstly, they would get feedback throughout the test period about whose heart rate was higher. If the other participant’s heart rate was higher, the circle on the second screen would glow green; if their heart rate was comparable, the circle would glow white; and if their heart rate was lower, the circle would glow red. The second implication would be that if the participant achieved better results—regarding processing time and number of errors—they would be paid €10. Participants were then asked to make their final selection regarding the multitasking or the alternative condition. They were informed that they could switch between the two conditions or terminate their participation in the study at any time without negative consequences. Participants then filled out a written consent form.

Listening to white noise can generally be considered self-harming behavior [25]. During the study, over a period of 420 s, for reasons of standardization, the volume increased independently of the participant’s heart rate to the maximum of 75 dB. The colored circle procedure was also standardized, such that the circle glowed green for the first seven seconds, then white for a further three seconds, and finally red until participants finished the task. The idea was to reinforce the participants’ perception of their enhanced stress level and thus self-harming behavior during the task. No debriefing took place at the end of the test to avoid word spreading among students that a €10 reward would be received whether or not participants applied white noise. After completion of the study, participants were fully debriefed and all received €10, as the comparison was fictive.

### 2.4. Statistical Analyses

Statistical analyses were performed using R [44] and RStudio [45]. Structural equation modelling (SEM) using the lavaan package [46] was used to test a series of models and the main research questions. First, measurement models for each dark trait based on four item parcels were tested [47]. In the next step, three different structural models reflecting different conceptualizations of the Dark Triad’s nomological net were tested: Model 1 was a model with three correlated latent variables. Model 2 was a model in which only a general factor (the dark core) explained all indicators. Model 3 was a bifactor model. Model fit was judged using the χ^2^ test, CFI (0.90), RMSEA (0.08), and SRMR (0.05) [48,49]. Models were compared based on the χ^2^ difference test and the difference in CFI [50]. The best-fitting structural model was then selected, and the different dependent variables reflecting self-harming behaviors were entered. Data, code, and outputs can be found in an Open Science Framework (OSF) repository (https://osf.io/nqaek/?view_only=2f077a9c530c4952b7c7cf665b2b728c (accessed on 23 February 2021).

### 2.5. Results of Study 1

Findings revealed that 106 (70%) of the participants selected the multitasking variable in the first stage, 86 (57%) of the participants in the second stage, and 99 (66%) of the participants in the third stage (dependent variable in the following SEM). None of the participants asked to switch to the other condition or to terminate the study. The correlation between choosing the multitasking variant after listening to the white noise and after working on the AOSPAN exercises was 0.57 (*p* < 0.01), and the correlation between choosing the multitasking variant after working on the AOSPAN exercises and after introducing the confederate was 0.81 (*p* > 0.01). This indicates a stable behavioral choice.

#### 2.5.1. Model Fits

Fits for all measurement models as well as estimates of the construct reliabilities can be found in Table 2.

It can be seen that all measurement models exhibited good fit. The higher RMSEA values can be explained by the low number of df and should not be counted against the models [51].

Comparisons of the three different equation models are depicted in Table 3. The superior fit of Model 3 was reflected in the χ^2^-difference tests, which showed that Model 3 fitted significantly better than the other two models (all *p’s > 0*.05, see OSF for analyses and results).

**Figure 1:**
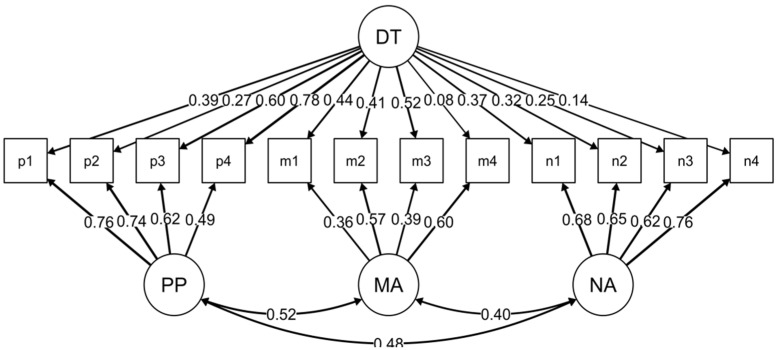
Structural equation model. DT = Dark Triad, PP = psychopathy, MA = Machiavellianism, NA = narcissism, p1–p4 = parcels for psychopathy, m1–m4 = parcels for Machiavellianism, n1–n4 = parcels for narcissism.

Figure 1 displays the structural equation model for Model 3 and thus the final model.

#### 2.5.2. Latent Regression

The bifactor model (dark core as well as the three traits as predictors) was used to estimate the impact of the Dark Triad core and traits on choosing multitasking as an indicator of self-harming behavior. The model had an acceptable model fit (χ^2^ [47] = 65.97, *p* = 0.035, CFI = 0.975, RMSEA = 0.052, SRMR = 0.046). The amount of explained variance was moderate, *R*^2^ = 0.094. However, in this model (Table 4), no individual predictor was significant.

## 3. Materials and Methods for Study 2

### 3.1. Sample, Informed Consent, and Ethical Approval

A total of 251 psychology students (76% female) with a mean age of 22.21 years (*SD* = 3.90) participated in the laboratory study. Informed consent was obtained from all participants. They were informed that their participation was anonymous, completely voluntary, and that they could terminate their participation in the study at any time without negative consequences. Participants were also informed that the collected information would only be used for the present study. Subjects were thanked and debriefed after participation. While planning the study, great care was taken to make sure that the study protocol adhered to the APA’s Ethical Principles of Psychologists and Code of Conduct. Specific attention was paid to the participants’ well-being in light of the treatments applied.

### 3.2. Measures

A different measure for assessing the Dark Triad was used in the second study because of the longer processing time but also because of the good psychometric properties of the Short Dark Triad (SD3) [30]. A German translation that had been shown to yield good psychometric properties [1] was administered. It consisted of 27 items, assessing the three subscales “narcissism” (α = 0.64; e.g., “People see me as a natural leader”), “psychopathy” (α = 0.66; e.g., “I’ll say anything to get what I want“) and “Machiavellianism” (α = 0.72; e.g., “Make sure your plans benefit you, not others“), with nine items each. Participants indicated their extent of agreement on a five-point rating scale, ranging from 1 (I strongly disagree) to 5 (I strongly agree).

For the deliberate self-harming behavior, the electric shock reaction game, ‘Lightning Reaction Reloaded’, was used. The game contains an electric shock device with four levels of intensity. Level 3 was exclusively used in the experiment as a pre-test with 15 students revealing that levels 1 and 2 were not perceived as different by participants.

### 3.3. Study Design

The study comprised an online questionnaire assessing the Dark Triad to be completed at home, as well as a laboratory test. Upon arrival, participants had to confirm that they did not meet exclusion criteria for the experiment based on the ‘Lightning Reaction Reloaded’ game instructions: no heart disease, epilepsy, or similar diseases and not currently pregnant. In the laboratory part of the experiment, we replicated a low-stimulus laboratory booth [18]. The booth measured approximately 4 m^2^ and had white, unadorned, soundproof walls. Inside, students found a table, a computer without a power connection, a chair, and the “Lightning Reaction Reloaded” game. No cell phones or similar devices were allowed. Participants were informed that the experiment would take approximately half an hour and that they would accomplish two consecutive tasks, each of which had a potential €5 reward.

#### 3.3.1. Neutral Condition

In the first condition, participants were told that they should stay in the booth for 10 min. Should they do so successfully, they would receive €5. However, participants were also given the opportunity to reduce the time they spent in the booth. They were given the option to self-administer one to five electric shocks, saving two minutes in the booth per shock. Using the electric shocker was considered to be self-harming behavior. Participants were assured that neither using nor ignoring the electric shocker would influence the course of the study. No information was given about how pleasant or unpleasant the shock might feel. However, participants were given the opportunity to test the electric shocker beforehand. Participants were given instructions regarding the use of the device. In order to trigger electric shocks, participants had to grasp a handle with one hand and could then activate the electric shock by clamping down on the handle. Since the device played loud music each time it was used, the number of self-administered electric shocks could be documented by the instructor sitting outside. The functionality of the device was checked daily. The instructor stopped the first task when the time was up.

#### 3.3.2. Competitive Condition

Participants were told that they had the chance to receive another €5 by taking part in a competition with another participant, to be assigned randomly, after completion of the study. Participants were informed that the competitor who shocked himself/herself more often would receive the €5. If both shocked themselves equally often, the reward would be split. No debriefing took place at that time to avoid word spreading among students that a €10 reward would be received whether or not participants used the electric shocker. After completion of the study, participants were debriefed and all received €10, as the comparison was fictive.

All descriptive statistics and correlations can be found in Table 5. It can be seen here that narcissism levels did not significantly relate to the number of shocks given in either condition. Psychopathy and Machiavellianism scores were significantly and positively related to the number of shocks in both conditions. This calls for multivariate analyses to identify possible specific effects and separate shared effects.

### 3.4. Statistical Analyses

Statistical analyses were performed using R [44] and RStudio [45]. We used the same analytical approach, first testing measurement and then structural models. The best-fitting structural model was used to integrate the different outcome variables (i.e., electric shocks self-administered in each condition).

### 3.5. Results of Study 2

Overall, 31.5% of participants in the first and 41% of participants in the second condition inflicted electric shocks on themselves. Descriptive statistics for the Dark Triad traits separately for those who inflicted and did not inflict electric shocks on themselves can be found in Table 6.

#### 3.5.1. Model Fits

All measurement models yielded an acceptable model fit (Table 7).

As in study 1, the bifactor model yielded the best fit (Table 8), which was again reflected in significant χ^2^-difference tests (see OSF for detailed results). It should be noted that the residual of one psychopathy parcel had to be fixed to 0 (see OSF).

#### 3.5.2. Latent Regressions

The dark traits and their core explained a total of 16.9% of the variance in electric shocks applied in the neutral and 16.8% in the competitor condition. Both models had acceptable fit (neutral condition: χ^2^ [51] = 99.08, *p* = < 0.001, CFI = 0.915, RMSEA = 0.065, SRMR = 0.059; competitor condition: χ^2^ [51] = 100.05, *p* = < 0.001, CFI = 0.915, RMSEA = 0.065, SRMR = 0.058). The regression weight pattern was similar and can be found in Table 9.

The results show that the dark core predicted the number of electric shocks in the neutral condition. For psychopathy, while the bivariate association was positive, a negative relation was observed for the *ß*-weight, which suggests a net suppression effect. The association between electric shocks and Machiavellianism remained positive but did not reach the set significance level. In the competitive condition, the dark core again predicted the number of electric shocks. Here, a specific significant and positive relation occurred for Machiavellianism, while the association with psychopathy which was, again, positive for the bivariate association, but negative for the *ß*-weight, therefore suggesting another net suppression effect, just missed the set level of significance.

## 4. General Discussion 

This research built on prior findings showing that aggression related to the Dark Triad can not only be directed towards others, but also manifest in self-harming behavior. The research’s aims were to test whether such Dark Triad-related self-harming behavior occurred across situations and could also be elicited without being the by-product of other-directed aggression. To this end, two experiments were conducted. While study 1 used a white noise paradigm, study 2 operationalized self-harm as the application of electric shocks. Importantly, the adverse situation features were chosen to relate to specific features of each dark trait. The first study revealed that in situations with upward comparisons, where aggression against others was not possible, self-harming behavior was only marginally related to narcissism. This is not in line with earlier work [8]. One reason for this could be that the white noise was not perceived as sufficiently unpleasant [24] to reflect self-harming without other-harming behavior [8] as defined here [26]. Another explanation why narcissism was only a small driver of deliberate self-harming behavior might be that the ego-threat condition was private and not public and thus could not threaten others’ perception of participants [11]. A third reason might be that we did not assess the vulnerable side of narcissism [52,53], which would be more likely to respond to ego threats. Thus, further research is needed to answer the question of whether self-harming behavior can be related to the Dark Triad traits in situations where aggression against others is not possible.

The second study revealed that self-harming behavior was stable over the two conditions and that psychopathy and Machiavellianism had specific relations, while the dark core was associated with self-harming behavior across situations. This showcases the vulnerability associated with the Dark Triad. Thus, these findings support the assumption that electric shocks seem to be more appropriate reflections of deliberate self-harming behavior than white noise. Moreover, these findings support the assumption and previous findings that the setting not only plays an important role in triggering aggressive behavior, but also in explaining it with respect to individual differences [15]. Following calls to broaden the scope of contextual factors beyond competitive tasks [11], being in a nondescriptive room triggered self-administered electric shocks. This behavior was related to the dark core but also to psychopathy. While this relation was positive when construct overlap was not controlled for, it became negative in the multivariate analysis. Thus, the specific differences unique to psychopathy predicted the application of fewer electric shocks. While such a suppression effect should be replicated, it is also worth hypothesizing about. A net suppression basically means that the suppressor (psychopathy) overlapped with another predictor (possibly the dark core) outside of the criterion. Thus, it suppressed criterion-irrelevant variance and enhances the other predictor’s relation with the test criterion. In other words, there was no specific negative relation between psychopathy and self-harming behavior. Instead, the suppression reflected controlling for irrelevant variance. At the very least, this indicates the necessity to control for the overlap between the dark traits. Another potential conclusion would be that the positive bivariate correlation for psychopathy reflected variance shared with Machiavellianism as well. The latter, however, still shared variance with self-harming behavior once the overlap was controlled for. Thus, the findings support the notion of a strong overlap between psychopathy and Machiavellianism but also lend support to the assumption of specific variance within each of the two constructs.

For people higher in Machiavellianism, it was assumed that the experimental setting would undermine their motives to engage in strategic thinking [30], establish social hierarchies or assert power [16], and exploit others when it is profitable [17], but also that it might address their material gain motivation [20]. However, the findings revealed only small and nonsignificant effects. Nevertheless, Machiavellians’ reward sensitivity [35] apart from the dark core seemed to be the reason for self-administering electric shocks in the monetary reward condition rather than their material gain motivation, as this was shared with people high in psychopathy [20].

Our findings also inform the debate regarding the separability of psychopathy and Machiavellianism as already hinted at. In study 2, we found a specific effect for Machiavellianism, while psychopathy acted as a suppressor. This reflects an overlap in psychological processes but potentially also underscores the existence of processes unique to each of the two traits, leading to specific behavioral manifestations. At the same time, it has to be stressed that even more focus should be placed on the dark core [10] with regard to inherent psychological processes.

In terms of study designs, it should be noted that both studies involved deceiving the participants: the confederate was fictitious for reasons of standardization. (Upward) comparisons were necessary in order to assess self-harming behavior in people scoring higher on narcissism [12]. In the first study, participant pairs were used where the partner was in fact a fictious confederate. If we had wanted to work with real pairs and realize the design, either one of the partners would always need to have a higher pulse or both pulses would have had to be similar. Since only the upward comparison was relevant, at least twice as many participants would have been required. Therefore, the design was implemented as described. In condition 2 of the second study, the deception was justifiable as participants received at least the monetary reward they expected, which was especially relevant for people high in psychopathy [20] and Machiavellianism [35]. The white noise operationalization is a commonly used paradigm in Dark Triad research [8], and the chosen decibel level was lower than the German limit for music players. We did not replicate the electric shock procedure by Wilson and colleagues [18] as we preferred to use a game which has been approved for use in Germany and has defined exclusion criteria. However, a culture-independent procedure should be applied in future research for comparability and replication. Unfortunately, for capacity reasons, the sample size of the first study deviated from the required sample size, therefore lowering statistical power. Furthermore, it would have been better to assess the Dark Triad with the same measures in both studies. In addition, only self-reports were considered. Finally, we did not randomly vary the two conditions in the second study.

## 5. Conclusions

In conclusion, our findings reveal that the malicious side of the Dark Triad is not only other-directed, but also self-directed in some situations. The Dark Triad traits differed in their responsiveness to different settings but could also be shown to elicit self-harming behavior over two conditions, thereby suggesting a vulnerable dark side. This vulnerable side has been rarely studied so far. The initial study [8] as well as the present findings can serve as a foundation for further exploring when and why one’s own costs subjectively outweigh one’s own benefit for the dark core [10] and the unique variance of its traits. The present findings indicate that it is worthwhile to dive deeper into the matter of non-competitive vs. competitive settings to explore [11] the amount, the nature (e.g., stressful), and the potential (e.g., multitasking vs. nondescript room) of self-harm-triggering settings and their associations with the dark traits. Through such work, we might learn more about the interactions between the dark traits, such as the separability of Machiavellianism and psychopathy observed in the second study. Thus, each Dark Triad member seems to be triggered by unique provocations [15] not only with respect to aggressive behaviors towards others, but also regarding self-aggressive behaviors, which should be further explored in order to contain self-harming behavior in the long term.

## Figures and Tables

**Table 1:** Means, standard deviations, and correlations with confidence intervals.

Variable	*M*	*SD*	1	2	3
1. Multitasking	0.66	0.48			
2. NA	1.37	0.16	0.15		
			[−0.01, 0.30]		
3. PP	2.24	0.35	0.06	0.51 **	
			[−0.10, 0.22]	[0.39, 0.62]	
4. MA	3.13	0.44	−0.01	0.38 **	0.57 **
			[−0.17, 0.15]	[0.23, 0.51]	[0.45, 0.67]

Note. *M* and *SD* are used to represent mean and standard deviation, respectively. Values in square brackets indicate the 95% confidence interval for each correlation. The confidence interval is a plausible range of population correlations that could have caused the sample correlation (Cumming, 2014). ** indicates *p* < 0.01. NA = narcissism, PP = psychopathy, MA = Machiavellianism.

**Table 2:** Model fits for all measurement models.

	Narcissism	Psychopathy	Machiavellianism
χ^2^	10.172	10.909	3.522
df	2	2	2
*p*	0.006	0.004	0.172
CFI	0.965	0.975	0.979
RMSEA	0.151	0.169	0.081
SRMR	0.036	0.03	0.036
Ω	0.82	0.9	0.72

Note. CFI = comparative fit index. RMSEA = root mean square error of approximation. SRMR = standardized root mean square residual. Ω = Weighted Ω.

**Table 3:** Fits for three different structural equation models.

	Model 1	Model 2	Model 3
χ^2^	80.65	209.77	51.24
df	51	54	39
*p*	0.005	<0.001	0.091
CFI	0.959	0.781	0.984
RMSEA	0.063	0.142	0.046
SRMR	0.053	0.101	0.043

Note. CFI = comparative fit index. RMSEA = root mean square error of approximation. SRMR = standardized root mean square residual.

**Table 4:** Regression weight patterns.

Predictor	*b*	*se*	*z*	*p*	CI_lower_	CI_upper_	*ß*
Narcissism	0.082	0.050	1.637	0.102	−0.016	0.180	0.172
Psychopathy	0.085	0.063	1.357	0.175	−0.038	0.209	0.180
Machiavellianism	−0.021	0.060	−0.348	0.728	−0.138	0.097	−0.044
Common Core	−0.063	0.056	−1.113	0.266	−0.174	0.048	−0.132

Note. CI = 95% confidence intervals, *b* = unstandardized regression weight, *se* = standard error, *z* = critical value, *ß* = standardized regression weight.

**Table 5:** Means, standard deviations, and correlations with confidence intervals.

Variable	*M*	*SD*	1	2	3	4
1. NA	3.05	0.51				
2. PP	1.93	0.50	0.35 **			
			[0.24, 0.46]			
3. MA	2.67	0.54	0.15 *	0.48 **		
			[0.02, 0.27]	[0.38, 0.57]		
4. shocks NC	1.20	1.96	0.08	0.25 **	0.25 **	
			[−0.05, 0.20]	[0.13, 0.36]	[0.13, 0.36]	
5. shocks CC	1.70	2.25	0.06	0.26 **	0.29 **	0.63 **
			[−0.07, 0.18]	[0.14, 0.37]	[0.17, 0.40]	[0.55, 0.70]

Note. *M* and *SD* are used to represent mean and standard deviation, respectively. Values in square brackets indicate the 95% confidence interval for each correlation. The confidence interval is a plausible range of population correlations that could have caused the sample correlation (Cumming, 2014). * indicates *p* < 0.05. ** indicates *p* < 0.01. NA = Narcissism, PP = Psychopathy, MA = Machiavellianism, NC = neutral condition, CC = competitive condition.

**Table 6:** Descriptive Statistics for the Dark Triad traits separately for those who inflicted and did not inflict electric shocks.

Sample	*n* (%)	Dark Triad					
		Narcissism		Psychopathy		Machiavellianism	
		*M*	*SD*	*M*	*SD*	*M*	*SD*
SH_1_	79 (31.5)	3.14	0.51	2.10	0.51	2.59	0.51
NSH_1_	172 (68.5)	3.01	0.51	1.80	0.48	2.59	0.54
SH_2_	103 (41)	3.07	0.52	2.05	0.48	2.82	0.51
NSH_2_	148 (59)	3.04	0.51	1.84	0.50	2.56	0.54

Note. SH = self-harm: applying electric shocks, NSH = no self-harm: not applying electric shocks, _1_ = neutral condition, _2_ = competitive condition.

**Table 7:** Model fits for all measurement models.

	Narcissism	Psychopathy	Machiavellianism
χ^2^	13.756	0.528	15.757
df	2	2	2
*p*	0.001	0.768	<0.001
CFI	0.872	1	0.936
RMSEA	0.159	<0.001	0.156
SRMR	0.036	0.03	0.056
Ω	0.63	0.69	0.78

Note. CFI = comparative fit index. RMSEA = root mean square error of approximation. SRMR = standardized root mean square residual. Ω = weighted Ω.

**Table 8:** Fits for three different structural equation models.

	Model 1	Model 2	Model 3
χ^2^	116.45	225.41	93.31
df	51	54	43
*p*	<0.001	<0.001	<0.001
CFI	0.882	0.693	0.909
RMSEA	0.074	0.117	0.071
SRMR	0.072	0.099	0.062

Note. CFI = comparative fit index. RMSEA = root mean square error of approximation. SRMR = standardized root mean square residual.

**Table 9:** Regression weight patterns.

Predictor	*b*	*se*	*z*	*p*	CI_lower_	CI_upper_	*ß*
NC							
Narcissism	−0.11	0.38	−0.29	0.77	−0.85	0.63	−0.02
Psychopathy	−1.60	0.73	−2.18	0.03	−3.03	−0.16	−0.24
Machiavellianism	0.70	0.38	1.84	0.07	−0.05	1.45	0.16
Common Core	3.55	1.33	2.66	0.01	0.94	6.16	0.29
CC							
Narcissism	−0.31	0.47	−0.66	0.51	−1.22	0.61	−0.05
Psychopathy	−1.47	0.80	−1.85	0.06	−3.04	0.09	−0.20
Machiavellianism	1.02	0.45	2.25	0.02	0.13	1.91	0.21
Common Core	4.06	1.55	2.61	0.01	1.01	7.10	0.29

Note NC = neutral condition, CC = competitive condition, CI = 95% confidence intervals, *b* = unstandardized regression weight, *se* = standard error, *z* = critical value, *ß* = standardized regression weight.

## Data Availability

The data presented in this study are openly available in the OSF repository (https://osf.io/nqaek/?view_only=2f077a9c530c4952b7c7cf665b2b728c (accessed on 23 February 2021).
